# Evaluation of the ribosomal DNA internal transcribed spacer (ITS), specifically ITS1 and ITS2, for the analysis of fungal diversity by deep sequencing

**DOI:** 10.1371/journal.pone.0206428

**Published:** 2018-10-25

**Authors:** Rui-Heng Yang, Jin-He Su, Jun-Jun Shang, Ying-Ying Wu, Yan Li, Da-Peng Bao, Yi-Jian Yao

**Affiliations:** 1 Key Laboratory of Edible Fungal Resources and Utilization (South), National Engineering Research Center of Edible Fungi, Key Laboratory of Agricultural Genetics and Breeding of Shanghai, Institute of Edible Fungi, Shanghai Academy of Agricultural Sciences, Shanghai, China; 2 Computer Engineering College, Jimei University, Xiamen, China; 3 State Key Laboratory of Mycology, Institute of Microbiology, Chinese Academy of Sciences, Beijing, China; USDA Forest Service, UNITED STATES

## Abstract

The nuclear ribosomal DNA internal transcribed spacer (ITS) has been widely used to assess the fungal composition in different environments by deep sequencing. To evaluate the ITS in the analysis of fungal diversity, comparisons of the clustering and taxonomy generated by sequencing with different portions of the whole fragment were conducted in this study. For a total of 83,120 full-length ITS sequences obtained from the UNITE database, it was found that, on average, ITS1 varied more than ITS2 within the kingdom *Fungi*; this variation included length and GC content variations and polymorphisms, with some polymorphisms specific to particular fungal groups. The taxonomic accuracy for ITS was higher than that for ITS1 or ITS2. The commonly used operational taxonomic unit (OTU) for evaluating fungal diversity and richness assigned several species to a single OTU even with clustering at 99.00% sequence similarity. The clustering and taxonomic capacities did not differ between ITS1 and ITS2. However, the OTU commonality between ITS1 and ITS2 was very low. To test this observation further, 219,741 pyrosequencing reads, including 39,840 full-length ITS sequences, were obtained from 10 soil samples and were clustered into OTUs. The pyrosequencing results agreed with the results of the *in silico* analysis. ITS1 might overestimate the fungal diversity and richness. Analyses using ITS, ITS1 and ITS2 yielded several different taxa, and the taxonomic preferences for ITS and ITS2 were similar. The results demonstrated that ITS2 alone might be a more suitable marker for revealing the operational taxonomic richness and taxonomy specifics of fungal communities when the full-length ITS is not available.

## Introduction

Deep sequencing technologies and DNA barcoding are being increasingly applied to catalog and classify biodiversity. With the advent of high-throughput sequencing techniques, also known as next-generation sequencing, it has become feasible to study fungal diversity for both recovery of huge numbers of sequences from different environmental samples and in-depth analyses of fungal diversity at the same time. The nuclear ribosomal DNA (nrDNA) internal transcribed spacer (ITS) has been widely used in both molecular systematics and ecological studies of fungi [1–6] and has been selected as the formal barcode marker for fungi [4, 7]. Because of its multicopy nature [8], the ITS allows easy amplification from samples containing low DNA concentrations. Furthermore, thousands of ITS sequences of different species are readily available from various online databases including UNITE [9] and the International Nucleotide Sequence Database (GenBank, EMBL and DDBJ), providing a large reference collection for taxonomic classification [10].

However, because of the read length limitations of pyrosequencing or Illumina sequencing, only part of the ITS region is usually used, e.g., ITS1 or ITS2. These subregions have been successfully applied in the characterization of fungal communities in some complex ecosystems by the most widely used high-throughput sequencing (including pyrosequencing and Illumina platforms) and have revealed unexpectedly high fungal diversities [1, 11–15]. The Illumina platforms, HiSeq and MiSeq, have become more widely used for the analysis of fungal composition by increasing the length of reads and reducing costs. ITS1 and ITS2 are likely to be the prime targets for the evaluation of fungal diversity through deep sequencing [16].

There are some controversies in the selection of markers for sequencing. Comparisons between ITS1 and ITS2 for fungal profiles have been assessed in a number of studies. The result that ITS1 was more variable than ITS2 was almost consistent [17–19]. Within the ITS region, ITS1 evolved more rapidly and has a more variable length than ITS2 [20]. Some reports revealed that ITS1 and ITS2 yielded similar clustering and taxonomic results [17, 18]. However, taxonomic resolution was not equal at different taxonomic levels in terms of taxon identification with ITS1 and ITS2 [17]. Bazzicalupo et al. [17] also found that ITS2 seemed more suitable for revealing the richness of operational taxonomic units (OTUs) in fungal communities. However, Mello et al. [21] and Wang et al. [22] reported that ITS1 was probably the best choice for the study of fungi or eukaryotic species. As pointed out by some researchers, e.g., Nilsson et al. [10], standardization of the selection of particular ITS subregions for sequencing requires further study. Some factors might result in the emergences of conflicts, including length, GC content, interspecific variations, and clustering and taxonomic preferences of the target genes. Some characteristics of ITS1 and ITS2 might be limitations of their use in the fungal community.

In previous studies [17–19, 21, 22], comparisons of ITS1 and ITS2 were conducted mainly based on ITS1 and ITS2 sequencing separately or extracting ITS1 and ITS2 from the ITS database. Not all the ITS1 and ITS2 were from the same ITS. In our study, ITS1 and ITS2, regardless of *insilico* or pyrosequencing datasets, were all extracted from the same ITS, which might be more suitable to evaluate different portions of the ITS for investigating fungal communities and to clarify the divergences between them. In addition, most of the studies were focused on the influence of alpha diversity and beta diversity. The composition of each cluster (contained sequences) was not considered. This study attempted to examine the similarity in clustering using different portions of ITS sequences from existing *in silico* databases and pyrosequencing data. We hypothesized that the capacity of placing sequences into OTUs was different between ITS1 and ITS2.

## Materials and methods

### *In silico* analysis

#### Database building

The UNITE database containing ITS sequences [9] was downloaded from the web site http://unite.ut.ee/repository.php, which included high-quality sequences from GenBank, EMBL and DDBJ. Only sequences with the complete ITS region and no ambiguous bases were retained for the analysis. The downloaded data formed the Fungi_*insilico*ITS database and was split into different groups at the phylum (*Ascomycota*, *Basidiomycota*, *Chytridiomycota*, *Glomeromycota*, *Zygomycota*) and subphylum (*Pezizomycotina*, *Taphrinomycotina*, *Saccharomycotina*, *Agaricomycotina*, *Pucciniomycotina*, *Ustilaginomycotina*) levels, forming a set of *insilico*ITS databases, as listed in S1 Table. The complete ITS1 and ITS2 sequences were extracted from each sequence using the program ITSx to create separate *insilico*ITS1 and *insilico*ITS2 databases for each phylum and subphylum (S1 Table). The full length ITS, ITS1 and ITS2 with full latin binomials were used for taxonomic resolution. All the resulting databases are listed in S1 Table.

### Pyrosequencing analysis

#### Soil sampling, DNA isolation, PCR amplification and pyrosequencing

Ten soil samples were collected from an experimental site (34°28′05.60″ N, 99°51′59.09″ E) dominated by alpine shrublands and meadows. Based on observations over the years by local inhabitants and survey teams regarding the presence or absence of stromata of *Ophiocordyceps sinensis*, the 10 samples were divided into 3 types: Os, *O*. *sinensis* present; NOs, *O*. *sinensis* absent; MP, mycelial pellicle with soil particles firmly wrapping the sclerotia of *O*. *sinensis* (covered by the larval skeleton). The soil cores were sampled from the top 20 cm using a stainless steel cylindrical drill with a diameter of 5 cm, and the samples were stored at –20°C in a portable electrical freezer. Even when unplugged, the portable freezer could maintain –20°C for up to 12 hours, such as on an airplane. After transport to the laboratory, the soil samples were passed through a 2-mm sieve to remove plant tissues, roots, rocks, etc., and were stored at –20°C prior to further experiments.

The total genomic DNA was extracted using the Powersoil DNA Isolation Kit (MoBio, Carlsbad, CA, USA) following the manufacturer's instructions. The DNA concentration was quantified on a NanoDrop spectrophotometer (Thermo Scientific, Worcester, MA, USA). The fungal ITS region was amplified using the primers ITS5 (5’‒GGAAGTAAAAGTCGTAACAAGG‒3’) and ITS4 (5’‒ATCCTCCGCTTATTGATATGC‒3’) [23]. The 5’ end of the primer ITS4 was tagged with a 6 bp barcode. The PCR mixtures were as follows: 4 μl of 5× FastPfu Buffer, 1 μl of each primer (5 μM), 2 μl of dNTP mixture (2.5 mM), 2 μl of template DNA and 10 μl of H_2_O. The thermocycling conditions consisted of an initial denaturation at 95°C for 2 min, followed by 30 cycles at 95°C for 30 sec, 55°C for 30 sec, 72°C for 30 sec and a final extraction at 72°C for 5 min. Three separate reactions were conducted to account for potentially heterogeneous amplification from the environmental template for each sample. The PCR products were purified using the AXYGEN Gel Extraction Kit and were quantified using a NanoDrop (Wilmington, DE). The PCR concentrations ranged from 0.80 ng/μl to 7.6 ng/μl using a NanoDrop.An equimolar mix of all three amplicon libraries was used for pyrosequencing. Each of the 3 mixed PCR products was diluted to 0.80 ng/μl for pyrosequencing. Pyrosequencing was performed with the primer ITS4 from the 5' end of the entire ITS on a 454 Life Sciences GS FLX system (Roche Applied Biosystems, Nutley, NJ, USA) at Allwegene Company (Beijing).

The raw sequencing reads were initially trimmed using MOTHUR [24] and the sequences were removed according to the criterions: shorter than 60 bp, quality score ≤ 30, contained ambiguous bases or did not exactly match to primer sequences and barcode tags. Full-length ITS, ITS1 and ITS2 sequences were extracted from the trimmed reads using the program ITSx [25] to form databases hereafter referred to as PyroITS, PyroITS1, and PyroITS2 (S1 and S3 Tables). All the sequences have been deposited in the NCBI Sequence Read Archive (SRA) under accession number SRP126914.

#### Length variability, clustering and taxonomy

The lengths of ITS, ITS1 and ITS2 (for both the *insilico* and Pyro databases) were calculated by Mothur. The sequences in each database were clustered into OTUs at the 91–99% similarity levels using UCLUST [26], and the clusters were saved as OTU files associated with the database. The Chao and Shannon fungal richness and diversity indices were calculated using Mothur. The taxonomy analysis was conducted using Blast against the UNITE database. The clustering tree was constructed from OTUs abundances in each sample using R. The significant differences between different groups were tested using ANOVA test.

The similarity of each OTU was assessed by checking the commonality of the sequences contained in the same OTU in the ITS, ITS1 and ITS2 databases of the same series. In order to find the same OTU in the three datasets, searching the representative sequence for one OTU in other two clustering results from other two databases was performed. The commonality analysis was conducted with the equation below:

$$A=(\sum_{0-n}m)/N$$

A: the similarity between the two databases

N: the number of reads in database 1

n: the number of OTUs in database 1

m: the number of identical sequences in the same OTU in database 1 and database 2

### Statistic tests

The significance of the differences between the identification success rates of ITS, ITS1 and ITS2 was tested using Fisher’s exact test. The significances for the differences between sequence lengths and GC content of ITS1 and ITS2 were tested using Student’s t-test. The commonality of OTUs at sequence similarities 91–99% was tested using comparing t-test. The different taxa presented in different groups (Os and Nos) were tested by Mann Whitney u test. Tests were carried out using R, and *P* ≤ 0.05 was considered statistically significant.

## Results

### *In silico* analysis

#### Databases

A total of 83,120 full-length ITS sequences from kingdom *Fungi* were obtained from the UNITE database. The sequences were separated into five phyla, i.e., *Ascomycota* (39,673 sequences), *Basidiomycota* (23,681 sequences), *Chytridiomycota* (296 sequences), *Glomeromycota* (5,626 sequences), *Zygomycota* (1,359 sequences) and unclassified fungi (12,485 sequences), according to the sequence annotation. The sequences belonging to *Ascomycota* contained several subphyla, i.e., *Pezizomycotina* (35,206 sequences), *Taphrinomycotina* (146 sequences), *Saccharomycotina* (2,407 sequences) and unclassified *Ascomycota* (1,914 sequences). The sequences belonging to *Basidiomycota* contained *Agaricomycotina* (20,522 sequences), *Pucciniomycotina* (1,951 sequences), *Ustilaginomycotina* (400 sequences) and unclassified *Basidiomycota* (808 sequences) (S1 Table). The databases for these sequences are hereafter referred to as *insilico*ITS, *insilico*ITS1, and *insilico*ITS2, with the appropriate prefixes designated by taxonomic grouping (S1 Table). The databases unclassified fungi, unclassified *Ascomycota* and unclassified *Basidiomycota* were not considered at phylum or subphylum levels.

#### Length variation of ITS1 and ITS2 in different groups

**Fig 1:**
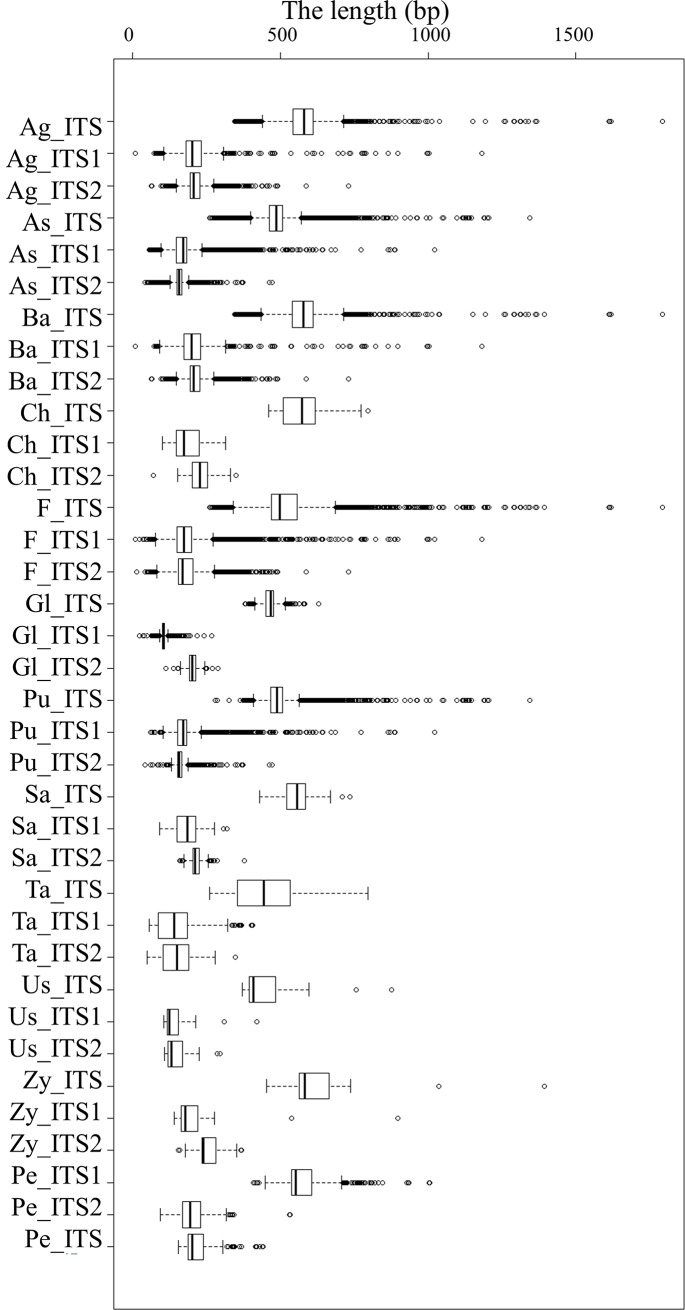
Length of ITS1 and ITS2 sequences in the major taxonomic groups at phylum and subphylum levels. As: Ascomycota; Pe: Pezizomycotina; Ta: Taphrinomycotina; Sa: Saccharomycotina; Ba: Basidiomycota; Ag: Agaricomycotina; Pu: Pucciniomycotina; Us: Ustilaginomycotina; Ch: Chytridiomycota; Gl: Glomeromycota; Zy: Zygomycota.

The length of the entire ITS ranged from 260 bp to 1,794 bp, with an average length of 517 bp (Fig 1, S1 Table). The ITS lengths in *Basidiomycota*, *Chytridiomycota* and *Zygomycota* were longer than those in *Ascomycota* and *Glomeromycota* (Fig 1, S1 Table). The ITS length was the shortest in subphyla *Taphrinomycotina* and *Saccharomycotina* (447 bp and 454 bp, respectively) (S1 Table).

ITS2 was longer than ITS1 in all the 5 phyla (*p*<0.001). The length of the extracted ITS1 portions ranged from 9 bp to 1181 bp, with an average length of 177 bp (S1 Table), and the length of the extracted ITS2 portions ranged from 14 bp to 730 bp, with an average length of 182 bp, among the fungi (S1 Table). At the subphylum level, ITS1 was longer than ITS2 in all subphyla of *Ascomycota* except for *Taphrinomycotina* (Fig 1B). Both ITS1 and ITS2 were shorter in *Ascomycota* than in the other fungal phyla (*Basidiomycota*, *Chytridiomycota*, *Glomeromycota* and *Zygomycota*, S1 Table). The size differential between ITS1 and ITS2 was greater in *Glomeromycota* than in *Ascomycota*, *Basidiomycota*, *Zygomycota* or *Chytridiomycota* (Fig 1A).

The length of ITS1 had a broader range of variation (S1 and S2 Tables). The sequences with a length longer than 600 bp or shorter than 100 bp were fewer in all the ITS2 datasets than in the Fungi kingdom, *Ascomycota*, *Basidiomycota*, *Zygomycota*, *Glomeromycota*, *Agaricomycotina*, *Pezizomycotina*, *Pucciniomycotina*, *Saccharomycotina* and *Ustilaginomycotina* ITS1 datasets (S2 Table). In *Chytridiomycota* and *Ustilaginomycotina*, the percentage of ITS2 was higher than that of ITS1. The highest rates of ITS1 and ITS2 were 28.08% and 23.68%, respectively, in Saccharomycotina.

#### GC content of ITS1 and ITS2 in different groups

**Fig 2:**
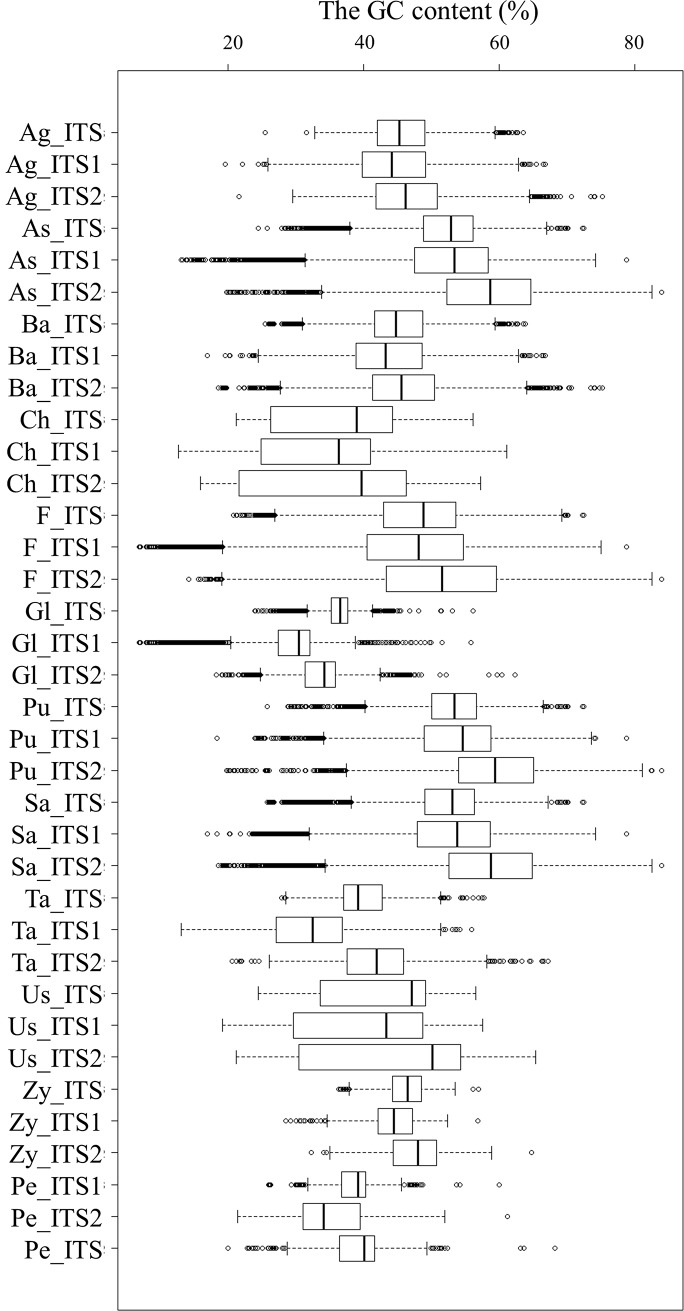
Box plots of GC content of ITS1 and ITS2 sequences in the major taxonomic groups. As: Ascomycota; Pe: Pezizomycotina; Ta: Taphrinomycotina; Sa: Saccharomycotina; Ba: Basidiomycota; Ag: Agaricomycotina; Pu: Pucciniomycotina; Us: Ustilaginomycotina; Ch: Chytridiomycota; Gl: Glomeromycota; Zy: Zygomycota.

The GC content of the ITS2 sequences was significantly higher than that of the ITS1 sequences in all the 14 major taxonomic groups (Fig 2). The mean GC contents of ITS1 and ITS2 were the highest in Ascomycota (52.31% and 57.76%, respectively), followed by Basidiomycota (43.49% and 46.06%, respectively), Zygomycota (35.14% and 38.92%, respectively), Chytridiomycota (33.97% and 36.19%, respectively) and Glomeromycota (28.87% and 33.93%, respectively) at phylum level. The GC contents of ITS1 and ITS2 were 53.85% and 589.00% in *Pezizomycotina*, 40.56% and 45.07% in *Taphrinomycotina* and, 31.86% and 41.91% in *Saccharomycotina*, respectively. And The GC contents of ITS1 and ITS2 were the highest in *Pucciniomycotina* (52.80% and 57.80%), followed by Agaricomycotina (44.44% and 46.57%) and Ustilaginomycotina (44.10% and 47.83%). The percentages of sequences with a GC content of less than 20% or greater than 80% in the *insilico*ITS2 and *insilico*ITS1 datasets were 0.27% and 0.96%, respectively (Fig 2). Less ITS2 sequences exceeded this threshold.

#### Fungal diversity and clustering commonality using different fragments

The fungal diversity and richness were evaluated by the Chao and Shannon indices. Clustering the different ITS portions from the Fungi_*insilico* databases Fungi_*insilico*ITS, Fungi_*insilico*ITS1 and Fungi_*insilico*ITS2 at 97% sequence similarity resulted in 16,554, 17,394, and 17,210 OTUs, respectively (S1 Table). Chao and Shannon indices were not significantly different between ITS, ITS1 and ITS2 (comparing t-test. *P*>0.05). However, the Chao and Shannon indices were higher in the Fungi_*insilico*ITS1 dataset (32,259 and 8.44, respectively) than in the Fungi_*insilico*ITS (30,788 and 8.35) and Fungi_*insilico*ITS2 (31,479 and 8.41) datasets. The same patterns of these diversity indices were presented in phyla Ascomycota, Zygomycota and in subphyla Saccharomycotina, Pucciniomycotina and Ustilaginomycotina. In contrast, the diversity and richness indices were higher in the ITS2 dataset for phylum Basidiomycota, Glomeromycota and Chytridiomycota and for subphyla Taphrinomycotina and Agaricomycotina (S1 Table).

**Fig 3:**
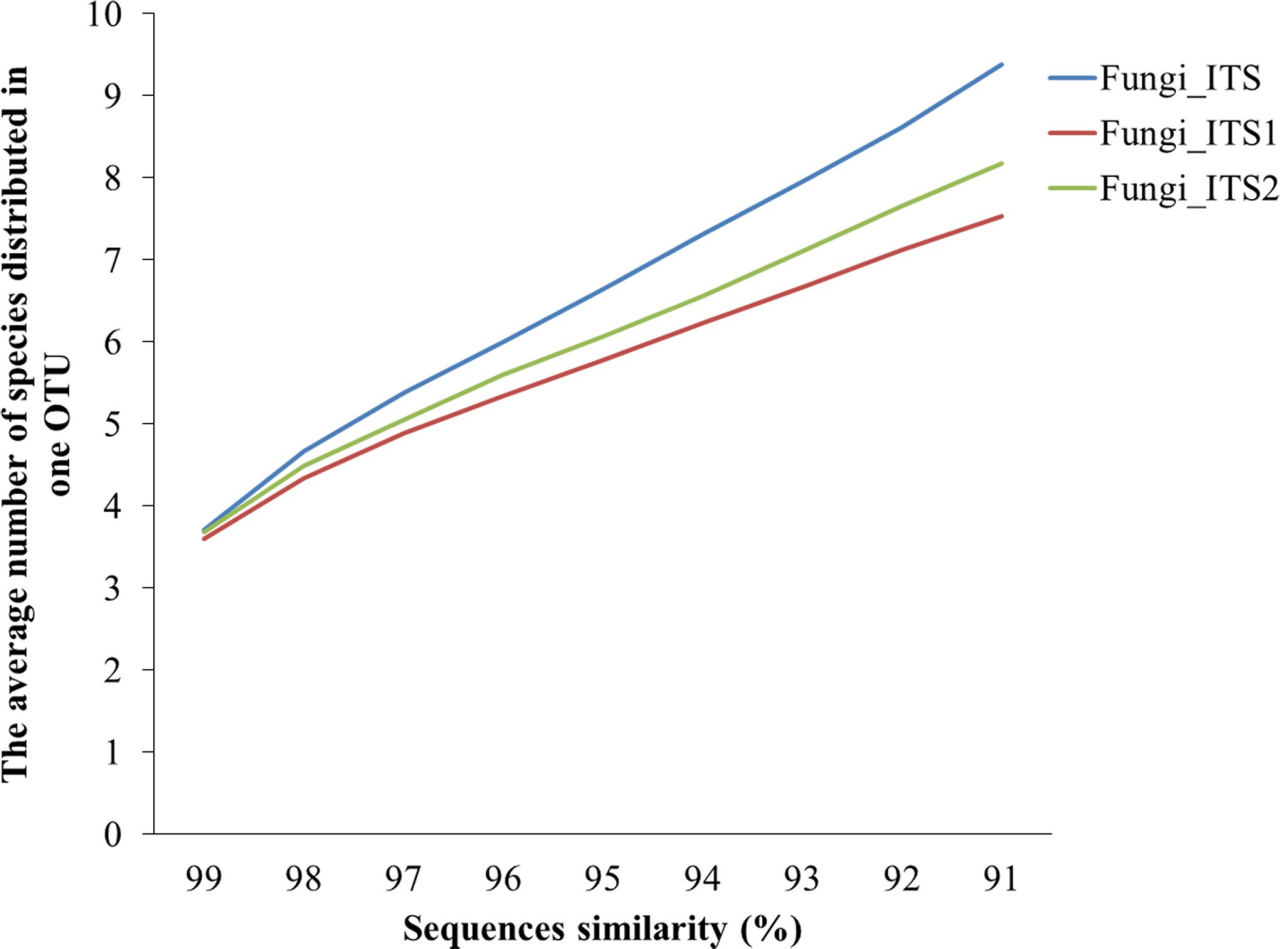
The number of species in one OTU in the Fungi_insilicoITS, Fungi_insilicoITS1 and Fungi_insilicoITS databases at 91–99% sequence similarity.

For the *insilico* databases, the results from the commonality analyses of the Fungi_*insilico*ITS1 and Fungi_*insilico*ITS databases were the same as those of the Fungi_*insilico*ITS2 and Fungi_*insilico*ITS databases (*p*>0.05, S2 Table). The similarity of Fungi_*insilico*ITS1 and Fungi_*insilico*ITS2 was 70–78% when clustered into OTUs at 97–98% sequence similarity (S3 Table). As seen in Fig 3, there were several species in one OTU. The average number of species in each OTU for ITS at 97% sequence similarity was 5.37, while the average number of species in each OTU for ITS1 and ITS2 were 4.89 and 5.05, respectively (Fig 3). Even at 99% sequence similarity, the three datasets (ITS, ITS1 and ITS2) had 3.71, 3.60 and 3.69 species, respectively, in one OTU (Fig 3). There were more different species in one OTU in the ITS dataset than in the ITS1 or ITS2 datasets.

#### Taxonomic resolution of ITS, ITS1 and ITS2

**Fig 4:**
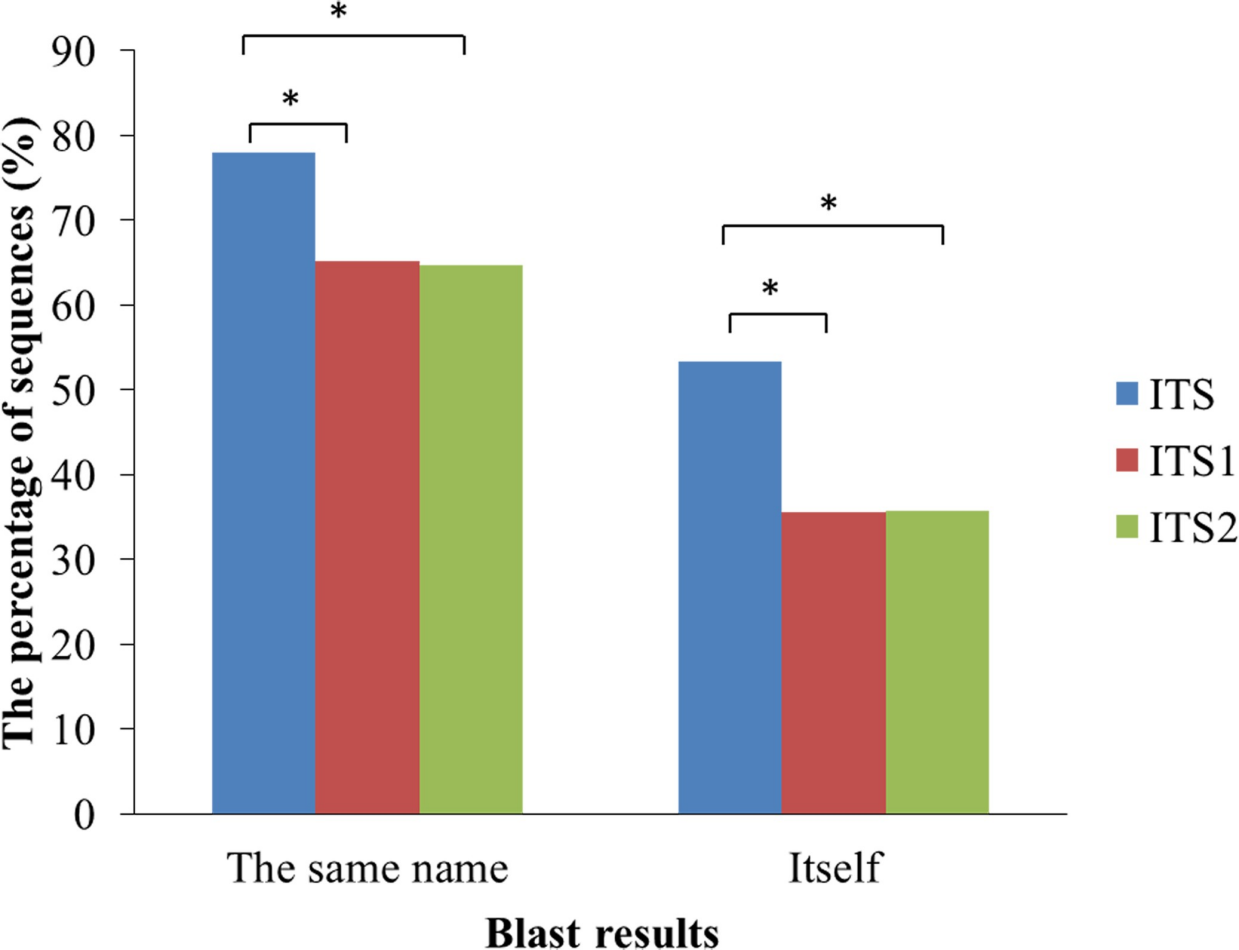
The sequence resolution obtained from blasting against the UNITE database.

Comparisons between the different portions of the ITS in terms of the resolution at which the reads were placed into taxa were also conducted. When blasted against the UNITE database, 53.27% of the ITS sequences returned themselves (the same accession number), which was a much higher percentage than for ITS1 (35.55%) and ITS2 (35.73%) (Fig 4). Taxonomic annotation is one of the most crucial steps in the identification of the fungal community, and it is important to annotate each sequence correctly. The queries used for blasting hit sequences with different accession numbers might belong to the same species. The number of the full length ITS sequences with full latin binomials (at species level) was 41,049 (The detailed information listed in S5 Table). The ITS analysis placed 78.00% of the queries into the same taxonomic groups, compared with only 65.16% and 64.72% for the ITS1 and ITS2 analyses, respectively (Fig 4). However, the resolution between ITS1 and ITS2 was not different in the placement of the reads into taxa. In addition, the same results were obtained at the phylum and subphylum levels.

### Pyrosequencing analysis

#### Pyrosequencing

A total of 219,741 reads were recovered from pyrosequencing, with various lengths ranging from 10‒747 bp. Among the reads, 34,398 were full-length ITS sequences.

#### Length variation and GC content of ITS1 and ITS2

For the 454 pyrosequencing data, the length of the extracted ITS1 fragments ranged from 58 bp to 278 bp, with an average length of 157 bp, and the length of the extracted ITS2 fragments ranged from 104 bp to 292 bp, with an average length of 158 bp. The length of ITS1 varied more than that of ITS2 in these reads. The GC content of the Pyro_ITS2 sequences was significantly higher (55.37%) than that of the ITS1 and ITS (50.35%) sequences.

#### Fungal diversity and richness in different samples

**Fig 5:**
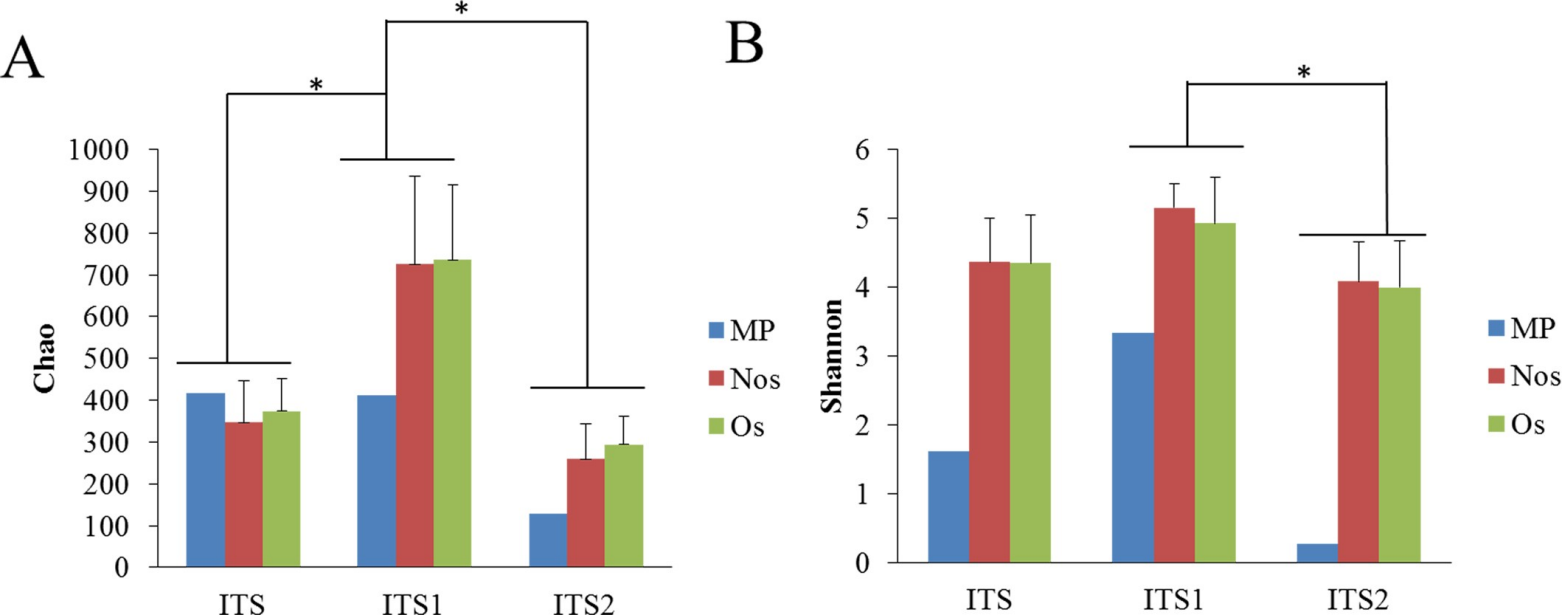
Fungal richness and diversity calculated based on ITS, ITS1 and ITS2. A: Chao index; B: Shannon index.

A total of 1377, 2895 and 1121 OTUs were generated from the PyroITS, PyroITS1, PyroITS2 databases, respectively, at 97% sequence similarity by UCLUST. The richness calculated based on the PyroITS1 database was much higher than that based on the PyroITS and PyroITS2 databases. The Chao indices were 347, 725 and 260 for the PyroITS, PyroITS1, and PyroITS2 databases, respectively (Fig 5). In addition, the Shannon diversity index was different between the PyroITS1 and PyroITS2 databases. The results revealed that the fungal diversity and richness might be overestimated when using ITS1 as the sequencing target.

**Fig 6:**
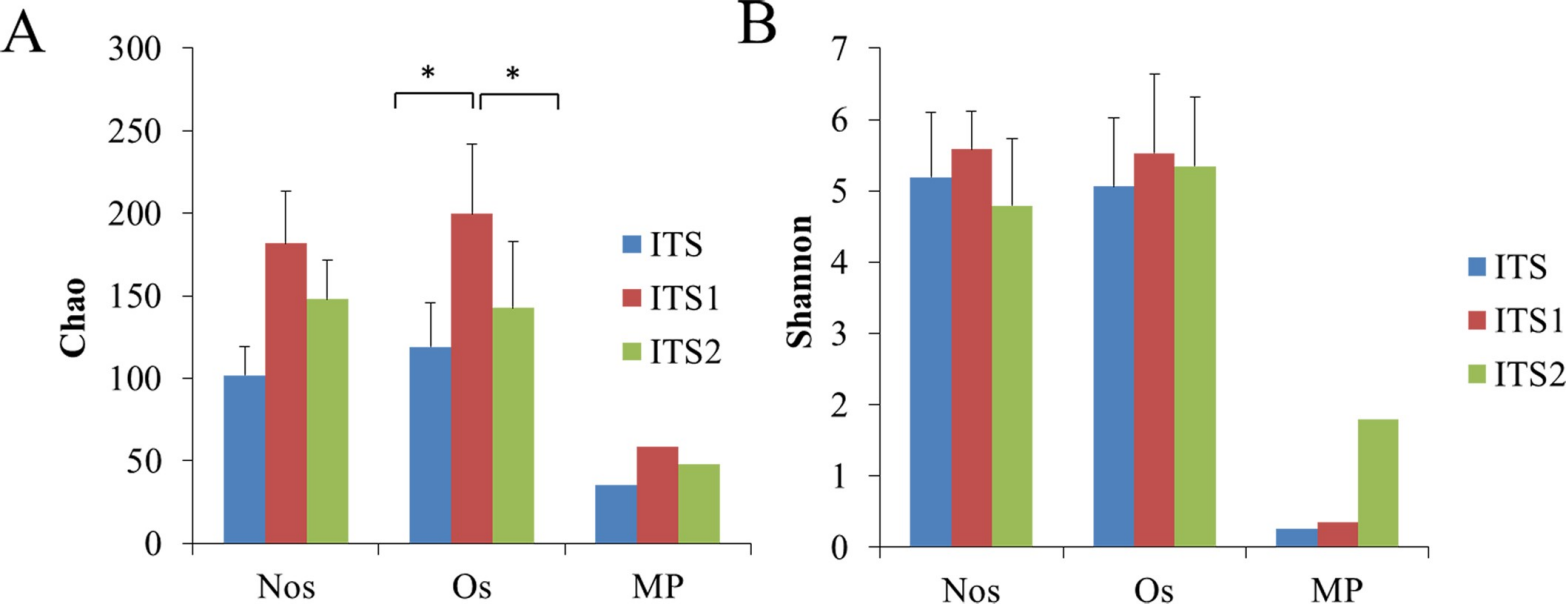
Fungal richness and diversity of Nos, Os and MP samples calculated based on ITS, ITS1 and ITS2. A: Chao index; B: Shannon index. Os: *O*. *sinensis* present; NOs: *O*. *sinensis* absent; MP: mycelial pellicle with soil particles firmly wrapping the sclerotia of *O*. *sinensis* (covered by the larval skeleton).

There were no differences between the Nos and Os type soils in terms of richness and diversity, and the richness and diversity of both of these were much higher than those of the MP type soils (Fig 6). The relationships between different samples in terms of diversity and richness were not influenced by the sequencing genes.

#### Cluster commonality using different fragments

For the pyrosequencing data, the assessment of the cluster similarity between the different databases (PyroITS, PyroITS1 and PyroITS2 databases, the sequences from the 10 samples pooled together) showed that the OTU commonality between PyroITS and PyroITS2 was higher (p<0.05), reaching 55.1% at 97% similarity, than that between PyroITS and PyroITS1 (26.8% at 97% similarity) (S6 Table) at 97% similarity. Furthermore, the commonality between the PyroITS1 and PyroITS2 databases at 97% similarity was only 28.2% (S7 Table).

**Fig 7:**
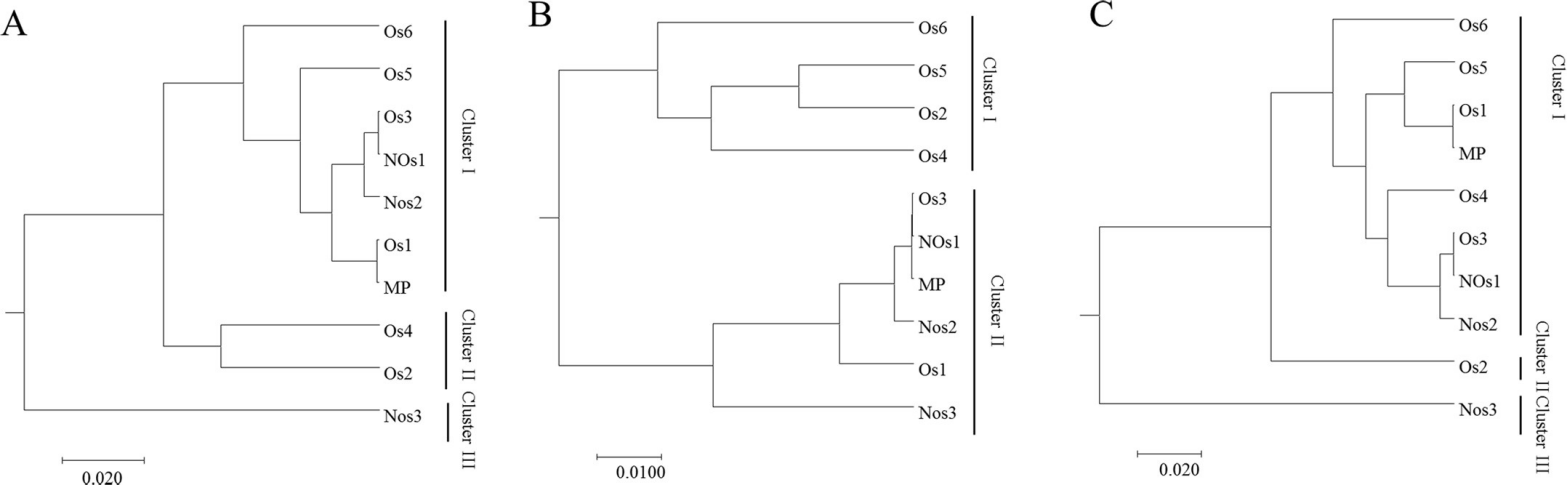
Hierarchical clustering analysis was performed based on the OTU compositions from PyroITS, PyroITS1 and PyroITS2. A: PyroITS; B: PyroITS1; C: PyroITS2. Os: *O*. *sinensis* present; NOs: *O*. *sinensis* absent; MP: mycelial pellicle with soil particles firmly wrapping the sclerotia of *O*. *sinensis* (covered by the larval skeleton).

A hierarchical clustering analysis was performed based on the OTU compositions of different samples. All three results revealed that the fungal beta-diversity in the different soils (Os, Nos and MP) was not different when analyzed with ANOVA. However, the details of the relationships between the samples were different. As shown in Figs 7 and 3 clusters were formed in PyroITS and PyroITS2. The differences mainly from one sample Os4. Os4 distributed in cluster II in PyroITS and OS4 in cluster I in PyroITS2. However, only 2 clusters formed in PyroITS1. Only 2 groups were clustered for ITS1 database: Os2, Os4, Os5 and Os6 clustered together; and Os1, Os3, Nos1, Nos3 and MP clustered together in the other branch (Fig 7). The clustering for ITS2 was much more similar to that for ITS than that for ITS1 (Fig 7).

#### The total fungal composition in the PyroITS, PyroITS1 and PyroITS databases

For ITS, ITS1 and ITS2, the percentage of the reads in the PyroITS database assigned to named taxa was higher (ranging from 99.28% at the phylum level to 59.21% at the genus level) than that in the PyroITS1 database (ranging from 92.50% at the phylum level to 59.54% at the genus level) or the PyroITS2 database (ranging from 92.90% at phylum level to 57.69% at genus level) (Table 1). At the phylum level, the reads belonging to Ascomycota and Basidiomycota accounted for more than 90% of the total sequences (S1 Fig). Dothideomycetes, Sordariomycetes, Leotiomycetes and Eurotiomycetes were the dominant classes in all the samples; in total, these classes represented 81.73%, 76.80% and 77.01% of the reads in the PyroITS, PyroITS1 and PyroITS2 databases, respectively (S1 Fig).

**Table 1:** Taxonomic information from the different pyrosequencing databases based on sequence.

		Phylum	Class	Order	Family	Genus
PyroITS	number of taxa	11	33	79	134	203
	Classified (%)	99.28	95.85	91.80	74.64	59.21
	unclassified (%)	0.72	4.15	8.20	25.36	40.79
PyroITS1	number of taxa	9	24	59	114	215
	classified (%)	92.50	87.84	84.53	69.30	59.54
	unclassified (%)	7.50	12.16	15.47	30.70	40.46
PyroITS2	number of taxa	8	24	59	114	205
	classified (%)	92.90	89.83	86.78	68.83	57.69
	unclassified (%)	7.10	10.17	13.22	31.17	42.31

Several different taxa were obtained when different target genes were used (ITS, ITS1 and ITS2), and the taxonomic preferences in the PyroITS and PyroITS2 databases were similar. After blasting against the UNITE database, 2, 3, 6, 12 and 34 different taxa were represented among the 3 databases at the phylum, class, order, family and genus levels, respectively (S7 Table). At the phylum level, more Chytridiomycota were targeted by ITS than by ITS1 or ITS2 (S7 Table). The percentages of Tremellomycetes in the PyroITS and PyroITS2 databases were 3.61% and 3.49%, respectively; these percentages were much higher than those in the PyroITS1 database (1.42%) at the class level. At the genus level, the percentages of *Peyronellaea* and *Microscypha* in the PyroITS database were 5.72% and 1.59%, respectively; these sequences were absent in the PyroITS1 and PyroITS2 databases. *Nectria*, *Dioszegia*, *Dactylonectria*, *Cladosporium* and *Holtermanniella* were nearly parallel in the PyroITS and PyroITS2 databases. The PyroITS1 database was more biased toward *Paraphoma* and Xenodidymella than were the PyroITS and PyroITS2 databases (S8 Table).

#### The fungal composition in different types of soil samples

After statistical analysis (mann whitney u test), the following taxa were found to be associated with Nos samples: in the PyroITS database, Rozellomycota_cls_Incertae_sedis at the class level, GS07 at the order level, and Comoclathris and *Tumularia* at the genus level (Table 2); in the PyroITS2 database, Pleomassariaceae at the family level; and in the PyroITS1 database, *Geomyces*, *Clavariopsis* and *Ophiosphaerella* at the genus level (Table 2). Auriculariales was possibly correlated with MP samples at the order level in the PyroITS2 database (Table 2). The taxa associated with Os samples were *Comoclathris* in the PyroITS and PyroITS2 databases and *Geomyces* in the PyroITS1 database (Table 2).

**Table 2:** Different taxa in the Nos, Os and MP samples based on ITS, ITS1 and ITS2.

			ITS		ITS1			ITS2		
	Taxa	Os	MP	NOs	Os	MP	NOs	Os	MP	NOs
Class										
	Rozellomycota_cls_Incertae_sedis	0.00	0.00	0.08	-	-	-	-	-	-
Order										
	GS07	0.00	0.00	0.08	-	-	-	-	-	-
	Auriculariales	-	-	-	-	-	-	0.00	0.10	0.00
Family										
	Lophiostomataceae	-	-	-	1.90	0.00	0.02	-	-	-
	Halosphaeriaceae	-	-	-	0.02	0.00	1.22	-	-	-
	Pleomassariaceae	-	-	-	-	-	-	0.07	0.00	1.22
Genus										
	*Ophiosphaerella*	0.00	0.00	0.13	-	-	-	-	-	-
	*Comoclathris*	2.60	0.00	0.05	-	-	-	2.40	0.00	0.05
	*Tumularia*	0.07	0.00	1.21	-	-	-	0.07	0.00	1.22
	*Geomyces*	-	-	-	1.29	0.00	0.14	-	-	-
	*Podospora*	-	-	-	0.00	0.00	0.00	-	-	-
	*Clavariopsis*	-	-	-	0.02	0.00	1.11	-	-	-
	*Ophiosphaerella*	-	-	-	0.00	0.00	0.10	-	-	-

Os: *O*. *sinensis* present; NOs: *O*. *sinensis* absent; MP: mycelial pellicle with soil particles firmly wrapping the sclerotia of *O*. *sinensis* (covered by the larval skeleton).

## Discussion

In this study, comparisons of clustering and taxonomy between different portions of the ITS were conducted based on online database (*in silico*) analyses and pyrosequencing reads (pyro). The results revealed that the clustering and taxonomy for ITS2 were more similar to those for ITS than to those for ITS1. The shorter length, lower GC content variation and greater taxonomic information content of ITS2 might make it more suitable than ITS1 for deep sequencing studies on fungal communities.

Some reports have shown that ITS1 and ITS2 generated similar patterns of community structure when used as DNA metabarcodes [17–19]. However, the commonality between ITS1 and ITS2 was very low in this study, especially in the pyrosequencing analyses; the similarity between the OTUs generated from the clustering of ITS1 and ITS2 at 97% similarity was only 28.20%. A possible explanation for the low similarity between ITS1 and ITS2 and the discrepancy from other studies [17–19, 21] is that the sequence compositions of each OTU were not considered, and ITS1 and ITS2 were sequenced separately. Another finding was that several species were present in one OTU [18]. In accordance with some fungal analysis methods, the representive sequences were used for taxonomic blasting. OTUs could be used to evaluate the fungal diversity in environmental samples. But, caution must be exercised when unveiling fungal community composition at the species level using ITS1 or ITS2.

In past years, there has not been consistent agreement about the selection of ITS1 or ITS2 in studies of fungal diversity. Nilsson et al. [27], Ihrmark et al. [28] and Alanagre et al. [29] stated that ITS2 was the better choice for 454 pyrosequencing or sequencing with Illumina platforms. However, Wang et al. [22] showed that ITS1 might be a better taxonomic DNA barcode than ITS2 in eukaryotes, based on *in silico* analyses. There are two possible explanations for this contradiction. First, the sequences used in Wang et al.’s study belonged to all eukaryotes, including fungi, plants and animals. In the present study, only fungal sequences were studied in detail, including *Ascomycota*, *Basidiomycota*, *Chytridiomycota*, *Glomeromycota*, *Zygomycota* and so on. Second, Wang et al. [22] mainly focused on species identification based on the annotations of the sequences stored in the NCBI database. In this study, not only were species identifications validated, but OTU clustering based on the ITS, ITS1 and ITS2 sequences was also considered; this method negated the effect of erroneous full Latin binomials.

In the present study, the intraspecific and interspecific variations of ITS1 were much higher than those of ITS and ITS2, in agreement with the results of previous studies [17, 18, 21, 27]. The clustering results obtained with ITS2 at 97% similarity might be the same as those obtained with ITS1 at 98 or 99% similarity.

As seen in the present study, the variation in length was greater for ITS1 than for ITS2 (Table 1, Fig 1) and more ITS1 sequences were shorter than 100 bp or longer than 600 bp; for example, most *Glomeromycota* ITS1 sequences were shorter than 100 bp. The length of ITS1 was more variable, likely due to the intron frequency [10, 20,27, 30]. The length variation and intron frequency might lead to unintended or inaccurate clustering or taxonomic placement [31]. In many studies, reads with a length of 100–150 bp are removed [18, 19]. These short reads that are filtered out might contain important taxonomic information. In addition, Illumina PE300 platforms can only cover sequences with a maximum length of 600 bp, longer sequences might not be overlapped in downstream analyses. More ITS2 sequences could pass through the filtering processing step.

It is usually difficult to amplify PCR products from templates with a high GC content compared to non-GC-rich templates [32]. The lowest GC content in the genomes of some species has been reported to be close to 20% [33, 34]. The GC content cutoffs were set at 20% and 80%. Although the GC content of ITS2 was slightly higher than that of ITS1, fewer sequences were filtered out with this criterion (<20% and >80%). ITS2 might have a positive effect on PCR and sequencing efficiencies.

In addition, some potential amplification biases might introduce by various commonly utilized ITS primers during amplification. An *in silico* study to evaluate PCR biases by different primers revealed that some of the ITS primers had a high proportion of mismatches relative to the target sequences (ITS1 or ITS2) and introduce taxonomic biases during PCR, e.g. the primers ITS1-F, ITS1 and ITS5 biased towards amplification of *Basidiomyceta*, whereas others, the primers ITS2, ITS3 and ITS4 biased towards *Ascomyceta* [35]. However, a new primer pair covered ITS2 region was designed in 2016, 5.8S-Fun and ITS4-Fun. Both of the primers had high coverage (nearly 100%) for Fungi but lower coverage for some other eukaryote [36]. The suitable primers made ITS2 to be a more accepted regions to study environmental samples.

This study highlights the issue that the clustering of ITS1 and ITS2 in different taxa is variable and might generate different results when the sequences of ITS subregions are used as DNA metabarcodes for deep sequencing studies on fungi. Careful attention must be devoted to the selection of sequencing markers and taxonomic processing. Classifications at the species levels are not recommended. ITS2 might be the most suitable marker for fungal diversity because of its shorter length, lower GC variation, greater abundance of references in public databases, broader selection of lineage-specific primers and longer portion of its length that can provide taxonomic information.

## Supporting information

S1 Fig Fungal composition in PyroITS, PyroITS1 and PyroITS2 databases at phylum level. (DOCX)

S1 Table Information about the databases used in this study and the OTUs, Chao richness estimation index and Shannon diversity index generated from the different databases. (DOCX)

S2 Table Commonality analysis between the Fungi_*insilico*ITS1 and Fungi_*insilico*ITS2 databases at 95‒99% similarity. (DOCX)

S3 Table Commonality analysis between the Fungi_*insilico*ITS1 and Fungi_*insilico*ITS databases at 95‒99% similarity. (DOCX)

S4 Table Commonality analyses representing the percentage of OTUs common to the PyroITS1, PyroITS2 and PyroITS databases at 95‒99% similarity. (DOCX)

S5 Table The number of sequences used for taxonomic resolution blasting against the UNITE database. (DOCX)

S6 Table The detailed information of the sequences with full length ITS (including ITS1 and ITS2) and full latin binomials. (DOCX)

S7 Table Commonality analyses representing the percentage of OTUs common to the PyroITS1 and PyroITS2 databases at 95%, 96%, 97%, 98%, and 99% similarity. (DOCX)

S8 Table Different taxa in the all the PyroITS, PyroITS1 and PyroITS2 databases. (DOCX)
