# Supplementary figures and images for: Evaluation of the ribosomal DNA internal transcribed spacer (ITS), specifically ITS1 and ITS2, for the analysis of fungal diversity by deep sequencing
Source: PLoS One. 2018 Oct 25;13(10):e0206428. doi: 10.1371/journal.pone.0206428 (PMC6201957; doi:10.1371/journal.pone.0206428)

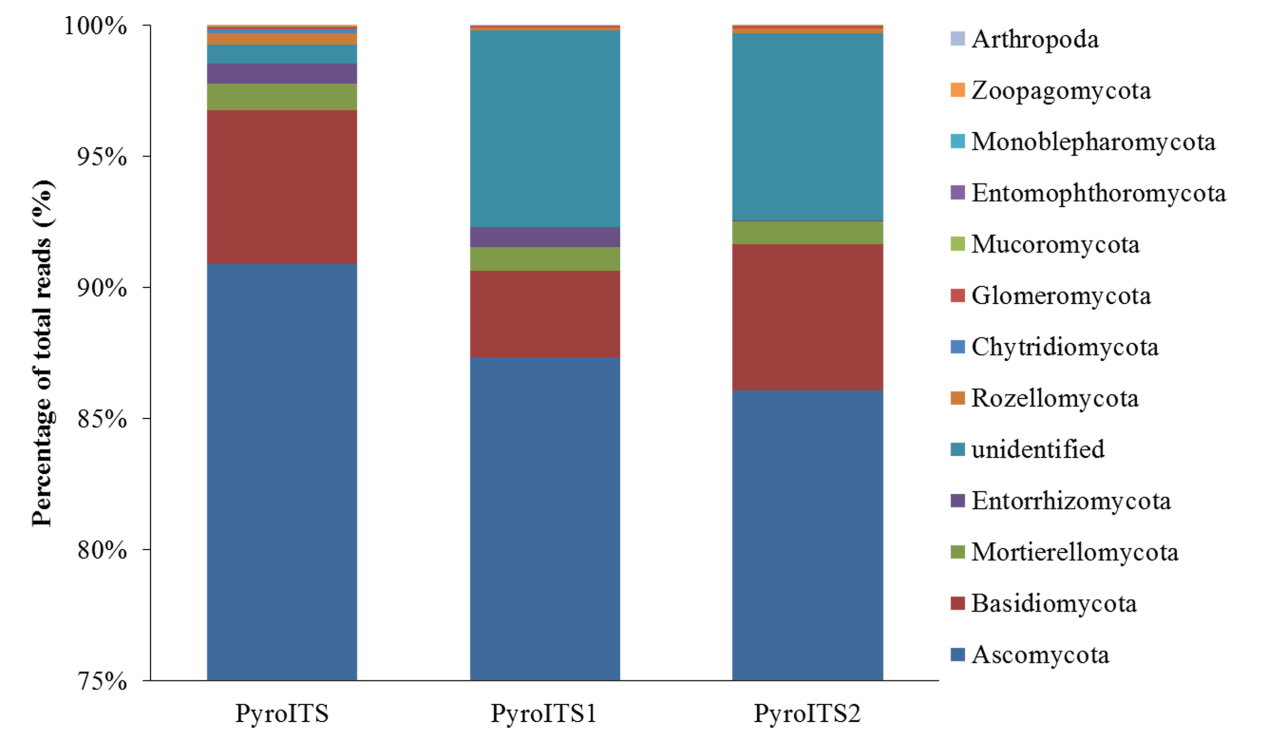


**S1 Fig. Fungal composition in PyroITS, PyroITS1 and PyroITS2 databases at phylum level.**

Supplement: S1 Fig — (DOCX) [file pone.0206428.s001.docx]
