# Supplementary material for: Evaluation of the ribosomal DNA internal transcribed spacer (ITS), specifically ITS1 and ITS2, for the analysis of fungal diversity by deep sequencing
Source: PLoS One. 2018 Oct 25;13(10):e0206428. doi: 10.1371/journal.pone.0206428 (PMC6201957; doi:10.1371/journal.pone.0206428)
Supplement: S1 Table — (DOCX) [file pone.0206428.s002.docx]

**S1 Table. Information about the databases used in this study and the OTUs, Chao richness estimation index and Shannon diversity index generated from the different databases.**

| Database | Range of sequence length (bp) | No. of sequences | Average length (bp) | OTUs | Chao | Shannon |
| --- | --- | --- | --- | --- | --- | --- |
| Fungi_*insilico*ITS | 260‒1794 | 83120 | 517 | 16554 | 30788 | 8.35 |
| Fungi_*insilico*ITS1 | 9‒1181 | 83120 | 177 | 17349 | 32259 | 8.44 |
| Fungi_*insilico*ITS2 | 14‒730 | 83120 | 182 | 17210 | 31479 | 8.41 |
| As_*insilico*ITS | 260‒1344 | 39673 | 490 | 6533 | 11841 | 7.27 |
| As_*insilico*ITS1 | 26‒1022 | 39673 | 172 | 7119 | 13010 | 7.35 |
| As_*insilico*ITS2 | 43‒473 | 39673 | 160 | 6865 | 12100 | 7.37 |
| Pe_*insilico*ITS | 279‒1344 | 35206 | 494 | 5624 | 9992 | 7.11 |
| Pe_*insilico*ITS1 | 62‒102 | 35206 | 174 | 6136 | 11089 | 7.16 |
| Pe_*insilico*ITS2 | 43‒473 | 35206 | 161 | 5969 | 10431 | 7.22 |
| Ta_*insilico*ITS | 372‒876 | 146 | 447 | 58 | 104 | 3.28 |
| Ta_*insilico*ITS1 | 56‒406 | 146 | 142 | 59 | 102 | 3.31 |
| Ta_*insilico*ITS2 | 50‒347 | 146 | 148 | 59 | 102 | 3.43 |
| Sa_*insilico*ITS | 260‒796 | 2407 | 454 | 402 | 702 | 4.75 |
| Sa_*insilico*ITS1 | 106‒421 | 2407 | 149 | 476 | 853 | 4.85 |
| Sa_*insilico*ITS2 | 109‒296 | 2407 | 147 | 396 | 709 | 4.59 |
| UA_*insilico*ITS | - | 1914 | - | - | - | - |
| UA_*insilico*ITS1 | - | 1914 | - | - | - | - |
| UA_*insilico*ITS2 | - | 1914 | - | - | - | - |
| Ba_*insilico*ITS | 345‒1794 | 23681 | 578 | 5862 | 10314 | 7.8 |
| Ba_*insilico*ITS1 | 9‒1181 | 23681 | 203 | 6147 | 10696 | 7.86 |
| Ba_*insilico*ITS2 | 63‒730 | 23681 | 214 | 6259 | 10939 | 7.9 |
| Ag_*insilico*ITS | 345‒1794 | 20522 | 577 | 5183 | 9187 | 7.68 |
| Ag_*insilico*ITS1 | 9‒1181 | 20522 | 205 | 5417 | 9531 | 7.73 |
| Ag_*insilico*ITS2 | 63‒730 | 20522 | 213 | 5534 | 9794 | 7.79 |
| Pu_*insilico*ITS | 279‒1344 | 1951 | 497 | 325 | 10385 | 7.19 |
| Pu_*insilico*ITS1 | 92‒320 | 1951 | 174 | 341 | 11421 | 7.28 |
| Pu_*insilico*ITS2 | 160‒378 | 1951 | 164 | 348 | 10858 | 7.3 |
| Us_*insilico*ITS | 453‒1394 | 400 | 609 | 106 | 171 | 3.92 |
| Us_*insilico*ITS1 | 142‒890 | 400 | 196 | 118 | 192 | 4.17 |
| Us_*insilico*ITS2 | 160‒378 | 400 | 257 | 110 | 169 | 3.94 |
| UB_*insilico*ITS | - | 808 | - | - | - | - |
| UB_*insilico*ITS1 | - | 808 | - | - | - | - |
| UB_*insilico*ITS2 | - | 808 | - | - | - | - |
| Ch_*insilico*ITS | 461‒796 | 296 | 575 | 118 | 223 | 4.29 |
| Ch_*insilico*ITS1 | 102‒314 | 296 | 188 | 119 | 229 | 4.32 |
| Ch_*insilico*ITS2 | 70‒351 | 296 | 233 | 125 | 275 | 4.35 |
| Gl_*insilico*ITS | 381‒629 | 5626 | 466 | 868 | 1360 | 5.5 |
| Gl_*insilico*ITS1 | 24‒269 | 5626 | 105 | 819 | 1184 | 5.48 |
| Gl_*insilico*ITS2 | 113‒289 | 5626 | 204 | 895 | 1350 | 5.65 |
| Zy_*insilico*ITS | 410‒1007 | 1359 | 578 | 236 | 407 | 4.2 |
| Zy_*insilico*ITS1 | 95‒533 | 1359 | 204 | 248 | 428 | 4.23 |
| Zy_*insilico*ITS2 | 154‒443 | 1359 | 216 | 250 | 423 | 4.42 |
| UF_ *insilico*ITS | - | 12485 | - | - | - | - |
| UF_ *insilico*ITS1 | - | 12485 | - | - | - | - |
| UF_ *insilico*ITS2 | - | 12485 | - | - | - | - |

As: Ascomycota; Pe: Pezizomycotina; Ta: Taphrinomycotina; Sa: Saccharomycotina; Ba: Basidiomycota; Ag: Agaricomycotina; Pu: Pucciniomycotina; Us: Ustilaginomycotina; Ch: Chytridiomycota; Gl: Glomeromycota; Zy: Zygomycota; UA: Unclassified Ascomycota; UB: Unclassified Basidiomycota; Un: Unclassified fungi.
