# Supplementary material for: Evaluation of the ribosomal DNA internal transcribed spacer (ITS), specifically ITS1 and ITS2, for the analysis of fungal diversity by deep sequencing
Source: PLoS One. 2018 Oct 25;13(10):e0206428. doi: 10.1371/journal.pone.0206428 (PMC6201957; doi:10.1371/journal.pone.0206428)
Supplement: S2 Table — (DOCX) [file pone.0206428.s003.docx]

**S2 Table. The number or percentage of sequences with length <100 bp or length >600.**

|  |  | The total number of sequences | The number or percentage of sequences with length <100 bp. No./(%) | The number or percentage of sequences with length >600 bp. No./(%) | Total  No./(%) |
| --- | --- | --- | --- | --- | --- |
| Fungi | ITS1 | 83120 | 1822 (2.19) | 85 (0.1) | 1907 (2.29) |
|  | ITS2 | 83120 | 611 (0.74) | 2 (0.00) | 613 (0.74) |
| As | ITS1 | 39673 | 828 (2.09) | 56 (0.14) | 884 (2.23) |
|  | ITS2 | 39673 | 580 (1.46) | 0 (0.00) | 580 (1.46) |
| Pe | ITS1 | 35206 | 140 (0.40) | 56 (0.16) | 196 (0.56) |
|  | ITS2 | 35206 | 6 (0.02) | 0 (0) | 6 (0.02) |
| Ta | ITS1 | 146 | 0 (0) | 0 (0) | 0 (0) |
|  | ITS2 | 146 | 0 (0) | 0 (0) | 0 (0) |
| Sa | ITS1 | 2407 | 676 (28.08) | 0 (0) | 676 (28.08) |
|  | ITS2 | 2407 | 570 (23.68) | 0 (0) | 570 (23.68) |
| Ba | ITS1 | 23681 | 100 (0.42) | 28 (0.10) | 128 (0.52) |
|  | ITS2 | 23681 | 3 (0.01) | 2（0.01) | 5 (0.02) |
| Ag | ITS1 | 20522 | 87 (0.42) | 26 (0.13) | 113 (0.55) |
|  | ITS2 | 20522 | 3 (0.01) | 2 (0.01) | 5 (0.02) |
| Pu | ITS1 | 1951 | 6 (0.31) | 0 (0) | 6 (0.31) |
|  | ITS2 | 1951 | 0 (0) | 0 (0) | 0 (0) |
| Us | ITS1 | 400 | 0 (0) | 2 (0.5) | 2 (0.5) |
|  | ITS2 | 400 | 0 (0) | 0 (0) | 0 (0) |
| Ch | ITS1 | 296 | 0 (0) | 0 (0) | 0 (0) |
|  | ITS2 | 296 | 6 (2.03) | 0 (0) | 6 (2.03) |
| Gl | ITS1 | 5626 | 791(14.06) | 0 (0) | 791(14.06) |
|  | ITS2 | 5626 | 0 (0) | 0 (0) | 0 (0) |
| Zy | ITS1 | 1359 | 5 (0.37) | 0 (0) | 5 (0.37) |
|  | ITS2 | 1359 | 0 (0) | 0 (0) | 0 (0) |

As: Ascomycota; Pe: Pezizomycotina; Ta: Taphrinomycotina; Sa: Saccharomycotina; Ba: Basidiomycota; Ag: Agaricomycotina; Pu: Pucciniomycotina; Us: Ustilaginomycotina; Ch: Chytridiomycota; Gl: Glomeromycota; Zy: Zygomycota
