# Supplementary material for: Evaluation of the ribosomal DNA internal transcribed spacer (ITS), specifically ITS1 and ITS2, for the analysis of fungal diversity by deep sequencing
Source: PLoS One. 2018 Oct 25;13(10):e0206428. doi: 10.1371/journal.pone.0206428 (PMC6201957; doi:10.1371/journal.pone.0206428)
Supplement: S3 Table — (DOCX) [file pone.0206428.s004.docx]

**S3 Table. Commonality analysis between the Fungi_*insilico*ITS1 and Fungi_*insilico*ITS2 databases at 95‒99% similarity**

|  |  | Fungi_*insilico*ITS1 | | | | |
| --- | --- | --- | --- | --- | --- | --- |
|  |  | 95 | 96 | 97 | 98 | 99 |
|  | 95 | 73.05 | 68.97 | 60.48 | 59.78 | 48.92 |
|  | 96 | 72.07 | 73.93 | 70.37 | 64.66 | 52.81 |
| Fungi_*insilico*ITS2 | 97 | 64.96 | 71.08 | 75.46 | 69.6 | 57.98 |
|  | 98 | 59.12 | 65.11 | 70.15 | 75.12 | 65.06 |
|  | 99 | 49.29 | 53.85 | 59.07 | 64.16 | 72.86 |
