# Supplementary material for: Evaluation of the ribosomal DNA internal transcribed spacer (ITS), specifically ITS1 and ITS2, for the analysis of fungal diversity by deep sequencing
Source: PLoS One. 2018 Oct 25;13(10):e0206428. doi: 10.1371/journal.pone.0206428 (PMC6201957; doi:10.1371/journal.pone.0206428)
Supplement: S4 Table — (DOCX) [file pone.0206428.s005.docx]

**S4 Table. Commonality analysis between the Fungi_*insilico*ITS1 and Fungi_*insilico*ITS databases at 95‒99% similarity**

|  |  | Fungi_*insilico*ITS | | | | |
| --- | --- | --- | --- | --- | --- | --- |
|  |  | 95 | 96 | 97 | 98 | 99 |
|  | 95 | 71.31 | 77.71 | 73.19 | 63.91 | 49.01 |
|  | 96 | 66.16 | 74.06 | 77.37 | 70.54 | 55.3 |
| Fungi_*insilico*ITS1 | 97 | 59.42 | 66.68 | 74.21 | 77.07 | 61.33 |
|  | 98 | 52.53 | 59.96 | 66.73 | 77.61 | 68.69 |
|  | 99 | 43.16 | 48.69 | 54.05 | 64.76 | 77.48 |
|  | 95 | 74.6 | 77.58 | 71.49 | 62.62 | 47.48 |
|  | 96 | 67.4 | 75.2 | 76.67 | 68.42 | 52.76 |
| Fungi_*insilico*ITS2 | 97 | 60.27 | 67.48 | 75.34 | 75.38 | 59.52 |
|  | 98 | 52.27 | 59.14 | 66.74 | 75.88 | 67.22 |
|  | 99 | 42.06 | 47.71 | 54.49 | 63.74 | 77.57 |
