# Supplementary material for: Evaluation of the ribosomal DNA internal transcribed spacer (ITS), specifically ITS1 and ITS2, for the analysis of fungal diversity by deep sequencing
Source: PLoS One. 2018 Oct 25;13(10):e0206428. doi: 10.1371/journal.pone.0206428 (PMC6201957; doi:10.1371/journal.pone.0206428)
Supplement: S5 Table — (DOCX) [file pone.0206428.s006.docx]

**S5 Table. The number of sequences used for taxonomic resolution blasting against the UNITE database.**

| Database | Range of sequence length (bp) |
| --- | --- |
|  |  |
| Fungi | 41049 |
| As | 26804 |
| Pe | 24915 |
| Ta | 64 |
| Sa | 1825 |
| Ba | 12325 |
| Ag | 10515 |
| Pu | 1478 |
| Us | 332 |
| Ch | 56 |
| Gl | 920 |
| Zy | 944 |

As: Ascomycota; Pe: Pezizomycotina; Ta: Taphrinomycotina; Sa: Saccharomycotina; Ba: Basidiomycota; Ag: Agaricomycotina; Pu: Pucciniomycotina; Us: Ustilaginomycotina; Ch: Chytridiomycota; Gl: Glomeromycota; Zy: Zygomycota
