# Supplementary material for: Evaluation of the ribosomal DNA internal transcribed spacer (ITS), specifically ITS1 and ITS2, for the analysis of fungal diversity by deep sequencing
Source: PLoS One. 2018 Oct 25;13(10):e0206428. doi: 10.1371/journal.pone.0206428 (PMC6201957; doi:10.1371/journal.pone.0206428)
Supplement: S6 Table — (DOCX) [file pone.0206428.s007.docx]

**S6 Table. Commonality analyses representing the percentage of OTUs common to the PyroITS1, PyroITS2 and PyroITS databases at 95‒99% similarity.**

| Database |  | PyroITS | | | | |
| --- | --- | --- | --- | --- | --- | --- |
|  | Similarity levels (%) | 95 | 96 | 97 | 98 | 99 |
|  | 95 | 33.5 | 37.1 | 41.6 | 48.0 | 29.0 |
|  | 96 | 31.6 | 33.8 | 33.9 | 44.5 | 34.1 |
| PyroITS1 | 97 | 19.4 | 21.9 | 26.8 | 31.8 | 37.3 |
|  | 98 | 15.1 | 16.7 | 22.5 | 25.1 | 41.9 |
|  | 99 | 9.5 | 10.2 | 13.1 | 16.2 | 29.2 |
|  | 95 | 79.9 | 64.4 | 47.7 | 37.0 | 20.1 |
|  | 96 | 75.3 | 71.5 | 52.4 | 41.2 | 21.3 |
| PyroITS2 | 97 | 69.6 | 68.9 | 55.1 | 42.3 | 22.4 |
|  | 98 | 45.6 | 56.7 | 54.7 | 43.6 | 25.3 |
|  | 99 | 47.9 | 55.0 | 64.5 | 45.9 | 29.7 |
