# Supplementary material for: Evaluation of the ribosomal DNA internal transcribed spacer (ITS), specifically ITS1 and ITS2, for the analysis of fungal diversity by deep sequencing
Source: PLoS One. 2018 Oct 25;13(10):e0206428. doi: 10.1371/journal.pone.0206428 (PMC6201957; doi:10.1371/journal.pone.0206428)
Supplement: S7 Table — (DOCX) [file pone.0206428.s008.docx]

**S7 Table. Commonality analyses representing the percentage of OTUs common to the PyroITS1 and PyroITS2 databases at 95%, 96%, 97%, 98%, and 99% similarity.**

| Database |  | PyroITS1 | | | | |
| --- | --- | --- | --- | --- | --- | --- |
|  | Similarity  Levels (%) | 95 | 96 | 97 | 98 | 99 |
|  | 95 | 44.3 | 38.1 | 26.0 | 22.0 | 14.7 |
|  | 96 | 45.9 | 38.5 | 27.3 | 22.4 | 15.0 |
| PyroITS2 | 97 | 49.7 | 42.8 | 28.2 | 23.2 | 16.0 |
|  | 98 | 47.7 | 42.6 | 30.7 | 25.8 | 17.3 |
|  | 99 | 48.9 | 42.3 | 34.1 | 29.0 | 20.9 |
