# Supplementary material for: Evaluation of the ribosomal DNA internal transcribed spacer (ITS), specifically ITS1 and ITS2, for the analysis of fungal diversity by deep sequencing
Source: PLoS One. 2018 Oct 25;13(10):e0206428. doi: 10.1371/journal.pone.0206428 (PMC6201957; doi:10.1371/journal.pone.0206428)
Supplement: S8 Table — (DOCX) [file pone.0206428.s009.docx]

**S8 Table. Different taxa in the all the PyroITS, PyroITS1 and PyroITS2 databases.**

|  | Taxa | ITS | ITS1 | ITS2 |
| --- | --- | --- | --- | --- |
| Class |  | 0.0000 | 0.0000 | 0.0000 |
|  | Glomeromycetes | 0.0622 | 0.0000 | 0.0475 |
|  | Rozellomycota_cls_Incertae_sedis | 0.0239 | 0.0000 | 0.0000 |
| Order |  |  |  |  |
|  | Holtermanniales | 0.7033 | 0.0000 | 0.7414 |
|  | GS07 | 0.0239 | 0.0000 | 0.0000 |
| Family |  | 0.0000 | 0.0000 | 0.0000 |
|  | Holtermanniales_fam_Incertae_sedis | 0.7033 | 0.0000 | 0.7414 |
|  | Trimorphomycetaceae | 0.1388 | 0.0000 | 0.1331 |
|  | Chrysozymaceae | 0.0670 | 0.0000 | 0.0333 |
|  | Microascaceae | 0.0478 | 0.0000 | 0.0190 |
| Genus |  |  |  |  |
|  | Peyronellaea | 5.7225 | 0.0000 | 0.0000 |
|  | Microscypha | 1.5885 | 0.0000 | 0.0000 |
|  | Nectria | 1.1818 | 0.0000 | 0.9601 |
|  | Holtermanniella | 0.7033 | 0.0000 | 0.7414 |
|  | Saitozyma | 0.1388 | 0.0000 | 0.1331 |
|  | Clavulinopsis | 0.0670 | 0.0000 | 0.0000 |
|  | Triscelophorus | 0.0574 | 0.0000 | 0.0000 |
|  | Gaeumannomyces | 0.0431 | 0.0000 | 0.0760 |
|  | Yunzhangia | 0.0335 | 0.0000 | 0.0000 |
|  | Rhexocercosporidium | 0.0287 | 0.0000 | 0.1663 |
|  | Pyricularia | 0.0239 | 0.0000 | 0.0000 |
|  | Rhinocladiella | 0.0191 | 0.0000 | 0.3660 |
|  | Fontanospora | 0.0000 | 0.0000 | 0.7319 |
| Phylum | Chytridiomycota | 0.1244 | 0.0048 | 0.0095 |
|  | Microbotryomycetes_ord_Incertae_sedis | 0.0766 | 0.0096 | 0.0380 |
|  | Acicuseptoria | 0.0000 | 0.0096 | 0.2186 |
|  | Dioszegia | 0.9474 | 0.0193 | 0.9030 |
|  | Cryptococcus | 0.4545 | 0.0386 | 0.2709 |
|  | Tremellaceae | 0.4976 | 0.0434 | 0.2899 |
|  | Inocybaceae | 0.1675 | 0.0530 | 0.0475 |
|  | Inocybe | 0.1675 | 0.0530 | 0.0475 |
|  | Gorgomyces | 0.0000 | 0.0579 | 0.0000 |
|  | Septoriella | 0.0335 | 0.0627 | 0.0000 |
|  | Capronia | 0.0048 | 0.0771 | 0.0048 |
|  | Annulatascales | 0.0000 | 0.1013 | 0.0000 |
|  | Annulatascaceae | 0.0000 | 0.1013 | 0.0000 |
|  | Pseudoproboscispora | 0.0000 | 0.1013 | 0.0000 |
|  | Sordariales_fam_Incertae_sedis | 0.0000 | 0.1302 | 0.0000 |
|  | Cordana | 0.0000 | 0.1302 | 0.0000 |
|  | Cadophora | 0.2057 | 0.1832 | 0.0856 |
|  | Cladosporium | 0.7512 | 0.2411 | 0.9743 |
|  | Mycosphaerella | 0.1435 | 0.3375 | 0.0000 |
|  | Amphisphaeriaceae | 0.0526 | 0.3423 | 0.7082 |
|  | Clavariopsis | 0.0000 | 0.3472 | 0.0000 |
|  | Mycosphaerellaceae | 0.1722 | 0.3761 | 0.0475 |
|  | Tremellales | 1.9522 | 0.4725 | 1.8821 |
|  | Nothophoma | 0.0000 | 0.5111 | 0.0000 |
|  | Endophoma | 0.0431 | 0.5400 | 0.0000 |
|  | Penicillium | 0.0909 | 0.6027 | 0.0713 |
|  | Dactylonectria | 0.8182 | 0.8004 | 0.0000 |
|  | Geomyces | 0.0000 | 0.8486 | 0.0048 |
|  | Hyaloscyphaceae | 3.3780 | 0.9161 | 1.1122 |
|  | Aspergillaceae | 0.4641 | 0.9402 | 0.4183 |
|  | Eurotiales | 0.4689 | 0.9450 | 0.4230 |
|  | Paraphoma | 0.4306 | 1.4176 | 0.0190 |
|  | Tremellomycetes | 3.6124 | 1.4176 | 3.4933 |
|  | Xenodidymella | 0.1340 | 1.4368 | 0.0000 |
|  | Didymella | 0.3158 | 4.3635 | 6.3878 |
